# Efficacy of Mecobalamin Tablets Combined with Troxerutin in the Treatment of NSCLC Chemotherapy-Induced Peripheral Neuropathy

**DOI:** 10.1155/2022/7946934

**Published:** 2022-09-26

**Authors:** Yang Li, Jiufu Gu, Qiquan Yu

**Affiliations:** ^1^Department of Thoracic, Longhua Hospital Affiliated to Shanghai University of Traditional Chinese Medicine, 200032 Shanghai, China; ^2^Department of Neurosurgery, Longhua Hospital Affiliated to Shanghai University of Traditional Chinese Medicine, 200032 Shanghai, China

## Abstract

**Objective:**

To assess the efficacy of mecobalamin tablets combined with troxerutin in the treatment of nonsmall cell lung cancer (NSCLC) chemotherapy-induced peripheral neuropathy (CIPN).

**Methods:**

From January 2020 to December 2021, 120 NSCLC patients with CIPN treated in our institution meeting the inclusion criteria were enrolled and assigned to receive mecobalamin tablets treatment in the control group, or assigned to receive mecobalamin tablets combined with troxerutin treatment in the research group, with 60 patients in each group. All patients were evaluated for clinical efficacy, neuropathic score, patient-reported CIPN symptoms, neuropathic pain grade, and quality of life after 3 weeks of treatment.

**Results:**

The clinical treatment effective rate of the patients in the research group was significantly higher than that of the patients in the control group (81.7% vs. 58.3%, *P* < 0.05). Compared with before treatment, neuropathic score, numbness and tingling score, hot/coldness in hands/feet score, and peripheral neurotoxicity grade in all patients decreased significantly after treatment (*P* < 0.05). And these reductions were more considerable in the research group compared to the control group (*P* < 0.05). In addition, the quality of life scores (EORTC QLQ-C30) increased significantly in all patients after treatment, and this rise was more considerable in the research group compared to the control group (*P* < 0.05).

**Conclusion:**

Mecobalamin tablets combined with troxerutin in the treatment of NSCLC patients with CIPN is effective and safe, and can significantly improve the symptoms and quality of life of NSCLC patients with CIPN.

## 1. Introduction

Lung cancer is one of the most common malignant tumors worldwide and can be divided into nonsmall cell lung cancer (NSCLC) and small cell lung cancer (SCLC), and NSCLC accounts for about 80% of all lung cancers [1]. Treatment of patients with NSCLC should be selected according to the size of intrapulmonary lesions, the size of extrapulmonary lesions, the location and number of metastases, and the patient's own situation. Surgery, chemotherapy, and radiation are the most common treatment protocols for patients with NSCLC [2]. About 75% of NSCLC patients are in the middle and advanced stages of the disease when they are first diagnosed, so they have lost the opportunity of surgical treatment, and the clinical treatment protocol mostly chooses chemotherapy. Paclitaxel and platinum drugs are the most commonly used chemotherapy drugs for patients with NSCLC [3, 4]. Although these chemotherapy drugs are of great significance in prolonging the survival time of patients and improving the treatment effect of patients, they can also cause sensory disturbances during clinical treatment and peripheral neurotoxicity, such as numbness in the hands and feet, which seriously affect the quality of life and chemotherapy tolerance of patients, thereby reducing the therapeutic effect of chemotherapy [5, 6]. Various drugs or supplements, including vitamin B1, glutathione, duloxetine, and venlafaxine, have been tested in clinical studies as an effective means of preventing the neurotoxicity of chemotherapy [7, 8]. However, most randomized controlled trials testing a variety of drugs with diverse mechanisms of action failed to reveal an effective treatment.

The mebendiamine tablet is a vitamin B12 preparation, and is mainly used for preventing and treating vitamin B12 deficiency, consumptive anemia, neuralgia, peripheral neuritis, and peripheral nerve paralysis.[9]. Previous studies have found that mecobalamin tablets were effective in treating disease or treatment-induced peripheral neuropathy, including diabetic peripheral neuropathy and FOLFOX4 chemotherapy-induced peripheral neuropathy (CIPN) [10, 11]. Troxerutin, also known as vitamin P4, is a derivative of the naturally occurring bioflavonoid rutin, found in tea, coffee, grains, and many vegetables and fruits [12]. Previous studies have shown that troxerutin is highly water-soluble, can be significantly absorbed by the gastrointestinal system, exerts protective effects without cytotoxic effects, and has been found to have antioxidant, anti-inflammatory, antidiabetic, and antitumor properties and other biological activities [13, 14]. Importantly, Gui et al. found that troxerutin alleviated chronic constriction injury (CCI)-induced neuropathic pain [15], but no studies have shown convincing evidence of substantial clinical benefit for troxerutin on the treatment of peripheral neuropathy. In the present study, we designed to study the efficacy of mecobalamin tablets combined with troxerutin in the treatment of NSCLC patients with CIPN.

## 2. Materials and Methods

### 2.1. Patients and Recruitment Criteria

The present study enrolled male and female adult NSCLC patients from January 2020 to December 2021, who were in remission after chemotherapy and presented with symptoms of CIPN. A total of 120 eligible patients with NSCLC were recruited to the study, and randomly divided into two groups, the control group and the research group, with 60 patients in each group. In the control group, there were 39 males and 21 females, aged from 40 to 72 years with an average age of (60.29 ± 5.38) years, 35 adenocarcinomas, and 25 squamous cell carcinomas. In the research group, there were 38 males and 22 females, aged from 40 to 70 years with an average age of (60.04 ± 6.01) years, 38 adenocarcinomas, and 22 squamous cell carcinomas. Studies involving human participants were reviewed and approved by the Ethics Committee of Longhua Hospital affiliated to Shanghai University of Traditional Chinese Medicine.

Inclusion criteria: (1) According to National Cancer Institute's Common Terminology Criteria for Adverse Event 4.0, patients were diagnosed with chemotherapy-related sensory and motor abnormalities such as numbness, tingling, burning, hyperesthesia, or weakness in the hands and feet; (2) Karnofsky *P* score >60 (The patient was able to take care of himself/herself for most of his/her life, but occasionally needed help from others. A higher KPS indicated that the patient was in better physical condition.); (3) Expected survival greater than 3 months; (4) Complete clinical data, including age, gender, weight and height, comorbidities, tumor TNM stage, tumor differentiation, chemotherapy protocol, and previous treatment protocol; (5) 18–75 years old; (6) Normal language and cognitive skills; (7) The grade being evaluated by Eastern Cooperative Oncology Group (ECOG) score standard was 0–2 (The patient can at least move freely and live independently, and can get up and move at least half of the time during the day.).

Exclusion criteria: (1) Neuropathy from any type of nerve compression; (2) Any mental disorder, such as depression, suicidal ideation, and bipolar disorder; (3) Intellectual disability or inability to communicate; (4) History of alcohol or drug addiction; (5) Abnormal liver or kidney function; (6) Peripheral nerve abnormalities caused by diabetes, arthritis, peripheral paresthesia, radiotherapy, and infection.

### 2.2. Treatment Protocols and Clinical Efficacy

Patients in the control group were treated with oral mecobalamin tablets (0.5 mg each time, 3 times a day, Weicai (China) Pharmaceutical Co., Ltd., H20143107), a total of 3 weeks of treatment. Patients in the research group were treated with oral mecobalamin tablets (0.5 mg each time, 3 times a day, Weicai (China) Pharmaceutical Co., Ltd., H20143107), and intramuscular troxerutin injection (0.12 g each time, 2 times a day, Changchun Haiyue Pharmaceutical Co., Ltd., H20060246), a total of 3 weeks of treatment.

### 2.3. Clinical Efficacy

The clinical efficacy of all patients was assessed according to the WHO anticancer drug common toxicity grading standard combined with the numerical rating sacle (NRS). The scores of paresthesia and functional status of limbs from mild to severe were 0–3 points, respectively. 0 point: no uncomfortable feeling; 1 point: mild paresthesia or paroxysmal, but no dysfunction; 2 point: moderate to severe paresthesia, mild limb dysfunction; 3 points: unbearable paresthesia and obvious limb dysfunction. The clinical efficacy was evaluated after 3 weeks of treatment. Significantly effective: peripheral neurotoxicity assessment decreased by ≥2 grades, or peripheral neurotoxicity assessment was grade 0; Efficient: peripheral neurotoxicity reduced by 1 grade; Invalid: peripheral neurotoxicity was not alleviated or aggravated.

### 2.4. Neuropathy Score

Neuropathy scores were scored on patients by two neurologists blinded to the study. As previously described [16], the neuropathy score was 15 points according to the NRS score. The higher the score was, the severer the lesion was. The neuropathy score totally included five parts, i.e., sensory symptoms (0–3 points), needle sensitivity (0–3 points), vibration threshold (0–3 points), strength (0–3 points), and deep tendon reflex (0–3 points).

### 2.5. Patient-Reported CIPN Symptoms

Before treatment and after 3 weeks of treatment, we evaluated patient-reported CIPN symptoms by FACT/GOG-NTX, including numbness, tingling, and hot/coldness in hands/feet. Items are scored from 0 to 4 (0 = not at all; 4 = very much) [17].

### 2.6. Quality of Life

Before treatment and after 3 weeks of treatment, we used EORTC Quality of Life Questionnare-Core 30 (QLQ-C30) scale to evaluate the quality of life in the patients. The EORTC QLQ-C30 scale includes 5 independent items and an overall health score, namely, the function of physical, role, emotional, cognitive and social, and total score [16]. The higher each score of QLQ-C30 was, the better the quality of life of patients would be.

### 2.7. Statistical Analysis

Data in the present study were analyzed by SPSS 20.0 software (SPSS Inc., Chicago, USA). Qualitative data are presented as counts (%), and *P*-values are calculated using chi-square or Fisher's exact test as appropriate. Kolmogorov–Smirnov test was used to check whether quantitative data conformed to a normal distribution, data that conformed to a normal distribution were presented as (mean ± standard deviation), and unpaired Student's *t*-test was used to compare differences and calculate *P*-values. Quantitative data that did not conform to a normal distribution are presented as the median (interquartile range), and Mann–Whitney *U* test was used to compare differences and calculate *P*-values.

## 3. Results

### 3.1. Baseline Characteristics

The baseline characteristics of patients including sex, nuclear status, age, BMI, TNM stage, tumor differentiation, tumor type, and previous treatment were not statistically different between the control and study groups (*P* > 0.05). There was comparability between the two groups of patients (Table 1).

### 3.2. Clinical Efficacy

After 3 weeks of treatment with different protocols in the two groups, we evaluated the clinical efficacy of all patients, and found that the clinical efficacy of patients in the control group was evaluated as significantly effective, efficient, and invalid in 10, 25, and 25 cases, respectively. Correspondingly, the clinical efficacy of patients in the research group was evaluated as significantly effective, efficient, and invalid in 15, 34, and 11 cases, respectively. The clinical treatment effective rate of the patients in the research group was significantly higher than that of the patients in the control group (81.7% vs. 58.3%, *P* < 0.05) (Table 2).

### 3.3. Peripheral Neurotoxicity Grade

There was no significant difference regarding peripheral neurotoxicity grade between the two groups before treatment, while significant difference was observed after treatment between the two groups with respect to peripheral neurotoxicity grade (*P* < 0.05) (Table3). In these two groups, peripheral neurotoxicity grade decreased considerably after treatment compared to before treatment. And such decrease which occurred in peripheral neurotoxicity grade in the research group was significantly stronger than that in the control group (*P* < 0.05) (Table 4).

### 3.4. Neuropathy Score

Before treatment, the neuropathy score of patients in the control group and the research group was (7.55 ± 2.12 points) and (7.60 ± 2.31 points), respectively, and there was no significant difference (*P* > 0.05). However, after 3 weeks of treatment with different protocols in the two groups, the neuropathy score of patients in the control group and the research group was all significantly decreased, and the neuropathy score of patients in the research group was significantly lower than that in the control group (3.05 ± 1.12 points vs. 3.53 ± 0.93 points, *P* < 0.05) (Table 5).

### 3.5. Patient-Reported CIPN Symptoms

Before treatment, the numbness and tingling score of patients in the control group and the research group was (2.00 ± 0.45 points) and (2.05 ± 0.52 points), respectively, and there was no significant difference (*P* > 0.05). However, after 3 weeks of treatment with different protocols in the two groups, the numbness and tingling score of patients in the control group and research group were all significantly decreased, and the numbness and tingling score of patients in the research group were significantly lower than that in the control group (1.00 ± 0.17 points vs. 1.55 ± 0.22 points, *P* < 0.05) (Table 3).

Before treatment, the hot/coldness in hands/feet score of patients in the control group and the research group was (1.95 ± 0.35 points) and (1.92 ± 0.41 points), respectively, and there was no significant difference (*P* > 0.05). However, after 3 weeks of treatment with different protocols in the two groups, the hot/coldness in hands/feet score of patients in the control group and the research group was all significantly decreased, and the hot/coldness in hands/feet score of patients in the research group was significantly lower than that in the control group (0.95 ± 0.12 points vs. 1.25 ± 0.18 points, *P* < 0.05) (Table 3).

### 3.6. Quality of Life

Before treatment, there was no significant difference in the quality of life between the two groups of patients being evaluated by the EORTC QLQ-C30 scale including the function of physical, role, emotional, cognitive and social, and total score (*P* > 0.05) (Table 6). After 3 weeks of treatment with different protocols, the quality of life assessed by the EORTC QLQ-C30 scale all changed in the two groups, and the score of physical, role, emotional, cognitive, and social function, and total score in the research group was all significantly higher than those in the control group (*P* < 0.05) (Table 7).

### 3.7. Adverse Effects

There were no treatment-related adverse reactions in both groups during the treatment period.

## 4. Discussion

Chemotherapy is an important protocol for the treatment of malignant tumors with remarkable curative effect, but chemotherapy drugs often indiscriminately damage normal cells in the process of destroying tumor cells. The sensory injury caused by the damage of peripheral nerves or autonomic nerves by chemotherapeutic drugs is called CIPN, and CIPN has a dose-limiting effect and is an important factor limiting the application of chemotherapeutic drugs. The toxic targets of chemotherapeutic drugs mainly include the central nervous system, peripheral nerves, and receptors, which are mainly manifested as symmetrical paresthesia, weakening, or loss of the extremities, and patients often feel burning, tingling, and paralysis [18, 19]. Platinum and third-generation cytotoxic drugs combined with chemotherapy are the first-line chemotherapy options for advanced NSCLC, but platinum chemotherapy drugs can easily lead to the appearance of CIPN in patients and affect the therapeutic effect. According to statistics [20, 21], the prevalence of CIPN was 68.1% in the first month after platinum-based chemotherapy, 60% in three months, and 30% of patients continued to suffer from peripheral neuropathy 6 months after chemotherapy.

In the present study, 120 NSCLC patients with CIPN were included in this study and divided into the control group and the research group according to their treatment methods. After 3 weeks of treatment in different ways, we found that the clinical treatment effective rate of the research group was 81.7%, which was significantly higher than the 58.3% clinical treatment effective rate of the control group. In addition, the improvement of neuropathic score, numbness and tingling score, hot/coldness in hands/feet score, and peripheral neurotoxicity grade in the research group were all significantly better than those in the control group. Therefore, these results indicated that the clinical efficacy of mecobalamin tablets combined with troxerutin in the treatment of CIPN in NSCLC patients is superior to that of mecobalamin tablets alone.

Adverse reactions of NSCLC chemotherapy patients are mainly divided into acute neurotoxicity and chronic neurotoxicity, mainly manifested as sensory loss and paralysis of peripheral nerves, with blood toxicity, neurotoxicity, hemoptysis, alopecia and other symptoms being the most common [22]. The onset of acute neurotoxicity is more rapid, mostly manifested as paresthesia and hypoesthesia, and chronic neurotoxicity is a delayed-onset peripheral neuropathy that occurs after multiple cycles of drug use, mainly manifested as deep sensory loss and psychomotor difficulties [23]. The occurrence of chronic neurotoxicity may be related to the following reasons. On the one hand, the neurotoxicity target of platinum drugs is in the spinal nerve followed by the ganglion, inhibiting ribosome synthesis in sensory neurons, blocking protein synthesis, and causing abnormal sensory neuron cell function which reacts with neurotoxicity [24]. On the other hand, oxidative stress, both calcium-magnesium mixtures and neuromodulators can be used as conventional drugs for the treatment of neurotoxic reactions, helping to restore the function of sensory nerve cells [25]. In addition, in our study, no significant adverse drug reactions were observed in the patients of the two groups, indicating that the combination of mecobalamin tablets combined with troxerutin in the treatment of peripheral neuropathy caused by NSCLC chemotherapy achieved good efficacy and high safety.

Mecobalamin is an endogenous coenzyme B12, mainly involved in the one-carbon unit cycle, and plays an important role in the transmethylation of homocysteine to methionine in the body. Modern drug research results have shown that mecobalamin can effectively promote the formation of neuronal myelin sheath and lecithin in the nervous system, thus generating a strong stimulation for axonal regeneration and nerve growth, and promoting its growth and development [26]. Therefore, mecobalamin was found to be effective in treating disease or treatment-induced peripheral neuropathy [9–11], which is consistent with the results of the present study. Troxerutin, also known as vitamin P4, has been shown to inhibit red blood cell and platelet aggregation, inhibit apoptosis, protect nerves, improve microcirculation, increase blood oxygen content, and promote angiogenesis [12, 27]. At present, troxerutin is mainly used for the treatment of edema, hemorrhoids, diabetic complications, venous thrombosis, cardiovascular, and cerebrovascular diseases, but its therapeutic efficacy in CIPN has not been studied [28, 29]. However, many studies have confirmed the neuroprotective effect of troxerutin, but the effect of troxerutin on CIPN is still unclear [30, 31]. Zhao et al. [30] found that troxerutin exerted neuroprotective effects in animal models of traumatic brain injury by regulating endothelial nitric oxide synthase coupling/uncoupling, and it can significantly reduce the nervous system damage, reduce the infarct size, and promote the integrity of the blood-brain barrier in animals with traumatic brain injury. In addition, previous studies have also revealed that trafluoretin can reduce the expression of glial fibrillary acidic protein and astrocyte DNA fragments and inhibit the loss of tyrosine hydroxylase-positive neurons in the substantia nigra, thus exerting neuroprotective effects in the mouse model of Parkinson's disease [31]. Although many studies have demonstrated neuroprotective properties of troxerutin, no studies have reported significant clinical benefit of troxerutin on the treatment of CIPN. In the present study, our results suggested that the clinical efficacy of mecobalamin tablets combined with troxerutin in the treatment of CIPN in NSCLC patients is superior to that of mecobalamin tablets alone.

## 5. Conclusion

Mecobalamin tablets combined with troxerutin in the treatment of NSCLC chemotherapy-induced peripheral neuropathy is effective and safe, and can significantly improve the symptoms and quality of life of NSCLC patients with CIPN. However, the present study is only a cohort study, the measurement indicators are highly subjective, and it fails to provide a causal conclusion, and does not conduct in-depth research on clinical communication skills, but it provides more ideas for in-depth research on the treatment of NSCLC chemotherapy-induced peripheral neuropathy in the future.

## Figures and Tables

**Table 1:** Baseline characteristics of the two groups in this study.

	Control group (*n* = 60)	Research group (*n* = 60)	*t*/*χ*^2^	*P*
Gender (*n*)				
Male	39	38	0.036	0.749
Female	21	22		

Age (years)	60.29 ± 5.38	60.04 ± 6.01	0.614	0.427

BMI (kg/m^2^)	22.67 ± 2.17	22.71 ± 2.24	0.725	0.305
Karnofsky performance status (points)	94.28 ± 7.41	94.88 ± 8.12	0.831	0.207

TNM stage (*n*)				
II	18	20	0.154	0.695
III–IV	42	40		

Differentiation (*n*)				
Low	8	9	0.170	0.918
Middle	30	31		
High	22	20		

Cancer type (*n*)				
Adenocarcinoma	35	38	0.315	0.575
Squamous cell carcinoma	25	22		

Previous treatment				
Previous surgery	41	39	0.150	0.699
Previous radiation therapy	6	7	0.086	0.769
Previous targeted therapy	5	4	0.120	0.729

**Table 2:** Comparison of clinical efficacy between the two groups of patients (*n* (%)).

Group	*n*	Significantly effective	Efficient	Invalid	Efficient rate
Control group	60	10 (16.7)	25 (41.7)	25 (41.7)	35 (58.3)
Research group	60	15 (25.0)	34 (56.7)	11 (18.3)	49 (81.7)
*χ* ^2^					7.778
*P*					0.005

**Table 3:** Comparison of neuropathic pain grade between the two groups of patients ($\bar{x}\pm s$, points).

Group	*n*	Numbness and tingling		Hot/coldness in hands/feet	
		Pretreatment	Posttreatment	Pretreatment	Posttreatment
Control group	60	2.00 ± 0.45	1.55 ± 0.22^*∗*^	1.95 ± 0.35	1.25 ± 0.18^*∗*^
Research group	60	2.05 ± 0.52	1.00 ± 0.17^*∗*^	1.92 ± 0.41	0.95 ± 0.12^*∗*^
*t*		0.625	5.328	0.779	4.265
*P*		0.408	0.011	0.284	0.042

*Note*. Compared with pretreatment, ^*∗*^*P* < 0.05.

**Table 4:** Comparison of peripheral neurotoxicity grade between the two groups of patients (*n*).

Group	*n*	Pretreatment			Posttreatment			
		1	2	3	0	1	2	3
Control group	60	18	20	22	13^*∗*^	26	17	4^*∗*^
Research group	60	12	24	24	23^*∗*^	29	6	2^*∗*^
*χ* ^2^		1.651			8.869			
*P*		0.438			0.031			

*Note*. Compared with pretreatment, ^*∗*^*P* < 0.05.

**Table 5:** Comparison of neuropathy score between the two groups of patients ($\bar{x}\pm s$, points).

Group	*n*	Pretreatment	Posttreatment
Control group	60	7.55 ± 2.12	3.53 ± 0.93^*∗*^
Research group	60	7.60 ± 2.31	3.05 ± 1.12^*∗*^
*t*		0.538	4.897
*P*		0.724	0.034

*Note*. Compared with pretreatment, ^*∗*^*P* < 0.05.

**Table 6:** Comparison of EORTC QLQ-C30 score before treatment ($\bar{x}\pm s$, points).

Group	*n*	Physical	Role	Emotional	Cognitive	Social	Total
Control group	60	72.4 ± 20.2	58.6 ± 24.4	63.2 ± 21.5	67.5 ± 23.0	66.7 ± 25.7	58.9 ± 12.4
Research group	60	74.5 ± 19.2	58.7 ± 26.7	64.8 ± 25.1	66.9 ± 25.2	67.4 ± 19.7	59.2 ± 13.0
*t*		0.698	0.997	0.718	0.921	0.807	0.439
*P*		0.301	0.168	0.299	0.172	0.212	0.638

**Table 7:** Comparison of *c* EORTC QLQ-C30 score after treatment ($\bar{x}\pm s$, points).

Group	*n*	Physical	Role	Emotional	Cognitive	Social	Total
Control group	60	79.9 ± 15.6	70.8 ± 17.4	68.7 ± 13.8	73.2 ± 19.1	78.6 ± 18.2	63.3 ± 10.2
Research group	60	84.8 ± 13.2	75.8 ± 18.2	72.6 ± 15.1	76.7 ± 18.7	83.4 ± 16.9	68.8 ± 9.4
*t*		6.992	7.428	6.057	5.972	7.897	9.630
*P*		0.002	<0.001	0.006	0.009	<0.001	<0.001

## Data Availability

The data used and/or analyzed during the current study are available from the corresponding author.

## References

[1] Siegel R. L., Miller K. D., Jemal A. (2020). Cancer statistics, 2020. *CA: A Cancer Journal for Clinicians*.

[2] Arbour K. C., Riely G. J. (2019). Systemic therapy for locally advanced and metastatic NSCLC: a review. *JAMA*.

[3] Nagasaka M., Gadgeel S. M. (2018). Role of chemotherapy and targeted therapy in early-stagenon-small cell lung cancer. *Expert Review of Anticancer Therapy*.

[4] Dafni U., Tsourti Z., Vervita K., Peters S. (2019). Immune checkpoint inhibitors, alone or in combination with chemotherapy, as first-line treatment for advanced non-small cell lung cancer a systematic review and network meta-analysis. *Lung Cancer*.

[5] Staff N. P., Grisold A., Grisold W., Windebank A. J. (2017). Chemotherapy-induced peripheral neuropathy: a current review. *Annals of Neurology*.

[6] Burgess J., Ferdousi M., Gosal D. (2021). Chemotherapy-induced peripheral neuropathy: epidemiology, pathomechanisms and treatment. *Oncology and Therapy*.

[7] Loprinzi C. L., Lacchetti C., Bleeker J. (2020). Prevention and management of chemotherapy-induced peripheral neuropathy in survivors of adult cancers: ASCO guideline update. *Journal of Clinical Oncology*.

[8] Hu L. Y., Mi W. L., Wu G. C., Wang Y. Q., Mao-Ying Q. L. (2019). Prevention and treatment for chemotherapy-induced peripheral neuropathy: therapies based on CIPN mechanisms. *Current Neuropharmacology*.

[9] Peng H. Y., Gong Y. Y. (2021). Analysis of the effect of probucol-mecobalamin tablets combination on oxidative stress in patients with diabetic peripheral neuropathy. *Neuroscience Letters*.

[10] Zhang Y., Fan D., Zhang Y. (2021). Using corneal confocal microscopy to compare mecobalamin intramuscular injections vs. oral tablets in treating diabetic peripheral neuropathy: a RCT. *Scientific Reports*.

[11] Li S. D., Shi J. H., Li X. J. (2016). Preventive effect of mecobalamin combined with glutathione on neurotoxicity induced by FOLFOX4 chemotherapy. *Zhonghua Zhongliu Zazhi*.

[12] Ahmadi Z., Mohammadinejad R., Roomiani S., Afshar E. G., Ashrafizadeh M. (2021). Biological and therapeutic effects of troxerutin: molecular signaling pathways come into view. *Journal of Pharmacopuncture*.

[13] Ghosh D., Dey S. K., Saha C. (2014). Antagonistic effects of black tea against gamma radiation-induced oxidative damage to normal lymphocytes in comparison with cancerous K562 cells. *Radiation and Environmental Biophysics*.

[14] Wang X., Gao Y., Wang L. (2021). Troxerutin improves dextran sulfate sodium-induced ulcerative colitis in mice. *Journal of Agricultural and Food Chemistry*.

[15] Gui Y., Li A., Chen F. (2015). Involvement of AMPK/SIRT1 pathway in anti-allodynic effect of troxerutin in CCI-induced neuropathic pain. *European Journal of Pharmacology*.

[16] Rostock M., Jaroslawski K., Guethlin C., Ludtke R., Schroder S., Bartsch H. H. (2013). Chemotherapy-induced peripheral neuropathy in cancer patients: a four-arm randomized trial on the effectiveness of electroacupuncture. *Evidence-Based Complementary and Alternative Medicine*.

[17] Smith E. M. L., Pang H., Cirrincione C. (2013). Effect of duloxetine on pain, function, and quality of life among patients with chemotherapy-induced painful peripheral neuropathy: a randomized clinical trial. *JAMA*.

[18] Bae E. H., Greenwald M. K., Schwartz A. G. (2021). Chemotherapy-induced peripheral neuropathy: mechanisms and therapeutic avenues. *Neurotherapeutics*.

[19] Zajączkowska R., Kocot-Kępska M., Leppert W., Wrzosek A., Mika J., Wordliczek J. (2019). Mechanisms of chemotherapy-induced peripheral neuropathy. *International Journal of Molecular Sciences*.

[20] Seretny M., Currie G. L., Sena E. S. (2014). Incidence, prevalence, and predictors of chemotherapy-induced peripheral neuropathy: a systematic review and meta-analysis. *Pain*.

[21] Bao T., Basal C., Seluzicki C., Li S. Q., Seidman A. D., Mao J. J. (2016). Long-termchemotherapy-induced peripheral neuropathy among breast cancer survivors: prevalence, risk factors, and fall risk. *Breast Cancer Research and Treatment*.

[22] Nurgalieva Z., Xia R., Liu C. C., Burau K., Hardy D., Du X. L. (2010). Risk of chemotherapy-induced peripheral neuropathy in large population-based cohorts of elderly patients with breast, ovarian, and lung cancer. *American Journal of Therapeutics*.

[23] Starobova H., Vetter I. (2017). Pathophysiology of chemotherapy-induced peripheral neuropathy. *Frontiers in Molecular Neuroscience*.

[24] Zhang S. (2021). Chemotherapy-induced peripheral neuropathy and rehabilitation: a review. *Seminars in Oncology*.

[25] Kachrani R., Santana A., Rogala B., Pawasauskas J. (2020). Chemotherapy-induced peripheral neuropathy: causative agents, preventative strategies, and treatment approaches. *Journal of Pain & Palliative Care Pharmacotherapy*.

[26] Zhang Y. F., Ning G. (2008). Mecobalamin. *Expert Opinion on Investigational Drugs*.

[27] Vidhya R., Anuradha C. V. (2020). Anti-inflammatory effects of troxerutin are mediated through elastase inhibition. *Immunopharmacology and Immunotoxicology*.

[28] Zamanian M., Bazmandegan G., Sureda A., Sobarzo-Sanchez E., Yousefi-Manesh H., Shirooie S. (2020). The protective roles and molecular mechanisms of troxerutin (vitamin P4) for the treatment of chronic diseases: a mechanistic review. *Current Neuropharmacology*.

[29] Qadiri A., Mirzaei Bavil F., Hamidian G. (2019). Administration of troxerutin improves testicular function and structure in type-1 diabetic adult rats by reduction of apoptosis. *Avicenna Journal of Phytomedicine*.

[30] Zhào H., Liu Y., Zeng J., Li D., Huang Y. (2018). Troxerutin cerebroprotein hydrolysate injection ameliorates neurovascular injury induced by traumatic brain injury-via endothelial nitric oxide synthase pathway regulation. *International Journal of Neuroscience*.

[31] Zhang S., Li H., Zhang L., Li J., Wang R., Wang M. (2017). Effects of troxerutin on cognitive deficits and glutamate cysteine ligase subunits in the hippocampus of streptozotocin-induced type 1 diabetes mellitus rats. *Brain Research*.

